# Grape Polyphenols Attenuate Diet-Induced Obesity and Hepatic Steatosis in Mice in Association With Reduced Butyrate and Increased Markers of Intestinal Carbohydrate Oxidation

**DOI:** 10.3389/fnut.2021.675267

**Published:** 2021-06-14

**Authors:** Esther Mezhibovsky, Kim A. Knowles, Qiyue He, Ke Sui, Kevin M. Tveter, Rocio M. Duran, Diana E. Roopchand

**Affiliations:** ^1^Department of Food Science and New Jersey Institute for Food, Nutrition, and Health (Rutgers Center for Lipid Research and Center for Nutrition, Microbiome, and Health), New Brunswick, NJ, United States; ^2^Department of Nutritional Sciences Graduate Program, Rutgers University, New Brunswick, NJ, United States

**Keywords:** hepatic steatosis, energy expenditure, *Akkermansia muciniphila*, butyrate, intestinal metabolism, Western diet, grape polyphenols, short chain fatty acids

## Abstract

A Western Diet (WD) low in fiber but high in fats and sugars contributes to obesity and non-alcoholic fatty liver disease (NAFLD). Supplementation with grape polyphenols (GPs) rich in B-type proanthocyanidins (PACs) can attenuate symptoms of cardiometabolic disease and alter the gut microbiota and its metabolites. We hypothesized that GP-mediated metabolic improvements would correlate with altered microbial metabolites such as short chain fatty acids (SCFAs). To more closely mimic a WD, C57BL/6J male mice were fed a low-fiber diet high in sucrose and butterfat along with 20% sucrose water to represent sugary beverages. This WD was supplemented with 1% GPs (WD-GP) to investigate the impact of GPs on energy balance, SCFA profile, and intestinal metabolism. Compared to WD-fed mice, the WD-GP group had higher lean mass along with lower fat mass, body weight, and hepatic steatosis despite consuming more calories from sucrose water. Indirect and direct calorimetry revealed that reduced adiposity in GP-supplemented mice was likely due to their greater energy expenditure, which resulted in lower energy efficiency compared to WD-fed mice. GP-supplemented mice had higher abundance of *Akkermansia muciniphila*, a gut microbe reported to increase energy expenditure. Short chain fatty acid measurements in colon content revealed that GP-supplemented mice had lower concentrations of butyrate, a major energy substrate of the distal intestine, and reduced valerate, a putrefactive SCFA. GP-supplementation also resulted in a lower acetate:propionate ratio suggesting reduced hepatic lipogenesis. Considering the higher sucrose consumption and reduced butyrate levels in GP-supplemented mice, we hypothesized that enterocytes would metabolize glucose and fructose as a replacement energy source. Ileal mRNA levels of glucose transporter-2 (GLUT2, *SLC2A2*) were increased indicating higher glucose and fructose uptake. Expression of ketohexokinase (*KHK*) was increased in ileum tissue suggesting increased fructolysis. A GP-induced increase in intestinal carbohydrate oxidation was supported by: (1) increased gene expression of duodenal pyruvate dehydrogenase (*PDH*), (2) a decreased ratio of lactate dehydrogenase a (*LDHa)*: *LDHb* in jejunum and colon tissues, and (3) decreased duodenal and colonic lactate concentrations. These data indicate that GPs protect against WD-induced obesity and hepatic steatosis by diminishing portal delivery of lipogenic butyrate and sugars due to their increased intestinal utilization.

## Introduction

A Western Diet (WD) characterized by excess calories from sugary, fat-laden foods and beverages promotes metabolic derangements such as obesity and non-alcoholic fatty liver disease (NAFLD) (1). Pharmaceutical approaches have been largely ineffective in curtailing the rising incidence of cardiometabolic disease; however, epidemiological studies suggest diets rich in polyphenol compounds may be a first line of defense. Dietary polyphenols demonstrate pleiotropic molecular activities that can target multiple symptoms of metabolic disease indicating these compounds have untapped therapeutic potential (2). B-type proanthocyanidins (PACs) are a major class of grape polyphenols (GPs) enriched in Concord grape seeds and skins. Despite their low bioavailability (3), PACs appear to mediate systemic effects *via* alteration of the gut microbiota (4–6). In association with profound changes to the gut microbial community, GPs improved glucose tolerance, reduced body weight gain, lowered levels of intestinal reactive oxidative species, and decreased intestinal inflammation in male mice fed a lard-based high fat diet (HFD) (4–6).

Mice fed a high-fat, high-sucrose (HFHS, based on soybean oil) diet had an increased relative abundance of Firmicutes and microbial genes that metabolize sugars into butyrate and acetate, which are lipogenic short chain fatty acids (SCFAs) (7). GP-supplementation of leptin receptor deficient (*db/db*) mice was found to reduce the relative abundance of *Clostridiales, Ruminococcaceae*, and *Lachnospiraceae* families in the Firmicutes phylum, which are major producers of SCFAs (8). PAC-rich extracts have been shown to reduce or alter SCFA profiles (9–13) while improving markers of metabolic health (11–13). SCFAs provide rodents and humans with ~5–10% of their daily energy requirements (14); therefore interventions that limit SCFA production may improve energy balance on a hypercaloric diet.

Short chain fatty acids have diverse functions, acting as both signaling and nutritive molecules. Butyrate is a major fuel source for ileal and colonic enterocytes (14, 15). Rodent and clinical studies have shown metabolic improvements after supplementation with fibers and/or polyphenols (13, 16–19) or pure SCFAs (20–22). Counterintuitively, higher SCFA concentrations were present in stool samples of obese (23–28), NAFLD (29) and hypertensive (30) patients. Furthermore, obesity-resistant mice showed resilience to diet-induced obesity and dyslipidemia, which was attributed to the reduced microbial production of SCFAs (31). Increased levels of fecal SCFAs suggests increased microbial energy harvest from the diet and thus an increased supply of intestinally derived nutrients to the liver and peripheral organs (28). Alternatively, increased concentrations of SCFAs in feces may suggest reduced intestinal SCFA absorption. The cardiometabolic health-benefits of PAC-rich extracts have been recapitulated in several studies (4–6, 8, 19, 32), therefore we hypothesized that a PAC-induced reduction in SCFA concentrations may be useful in the context of a hypercaloric diet. In this study we used a murine model of WD-induced metabolic disease to investigate the impact of a PAC-rich GP extract on energy balance, colonic SCFA levels, and intestinal metabolism.

## Materials and Methods

### Diets

As previously described (4, 5, 8), GPs were extracted from frozen Concord grape pomace (Welch Foods Inc., Concord, MA), quantified, and complexed to soy protein isolate (SPI, ProFam 955, ADM), to produce a GP-SPI complex containing 10% total polyphenols. This GP extract is rich (90%) in flavan-3-ol monomers (catechin and epicatechin) and PAC B2 dimers based on quantification by 4-(dimethylamino)cinnamaldehyde (DMAC) assay and LC-MS analysis (5). The nutritional composition of SPI and GP-SPI (Supplementary Table 1, Medallion Labs, Minneapolis, MN) was used by Research Diets (New Brunswick, NJ) to formulate a murine Western Diet (WD) or WD supplemented with GP (WD-GP) which delivered 1% GP (wt/wt) (Supplementary Table 2). Ingredient-matched and isocaloric WDs provided: (1) 46% of kilocalories (kcals) from fat derived from butter, corn oil, and cholesterol; (2) 39% kcals from carbohydrates (mainly sucrose); and (3) 15% kcal from protein (SPI and casein). The fatty acid composition of the butter in the WD is provided in Supplementary Table 3. The low-fat diet (LFD) provided (1) 15% of kcals from fat derived from butter and corn oil, (2) 70% kcals from carbohydrate (mainly corn starch), and (3) 15% kcals from protein. Mice fed WD or WD-GP were provided with autoclaved water containing 20% sucrose. Mice fed LFD were given autoclaved tap-water. Diet and drink were provided *ad libitum*.

### Animals and Metabolic Phenotyping

Experiments were performed with approval of Rutgers University Institutional Care and Use Committee. Five-week old male C57BL/6J mice (Cat# JAX:000664, Jackson Laboratory, Bar Harbor, ME) were housed in groups of 4–5 per cage and fed LFD for a 2-week acclimation period. To ensure diet groups started with equivalent oral glucose tolerance (OGT) at baseline, oral glucose tolerance tests (OGTT) were performed at age 7 weeks. Mice were then assigned to diet groups and single-housed in a temperature-controlled room (24 ± 1°C) inside ventilated cages with a 12:12 h (7 a.m.−7 p.m.) light-dark cycle. Each cage contained alpha-dry bedding and a shelter as enrichment. Mice (*n* = 6–8) received diets for 23 weeks. OGTT was repeated at 3, 12, and 23 weeks post-diet intervention. Body weights, food, and sucrose water intake were measured weekly. Total caloric intake and intake from carbohydrate, fat and protein was calculated using kcal% for each macronutrient based on the diet formulations provided by Research Diets (New Brunswick, NJ) (Supplementary Table 2). Body composition was analyzed by quantitative nuclear magnetic resonance imaging (EchoMRI 3-1 system, Echo Medical Systems, Houston, TX) at 7 and 21 weeks post-diet intervention, as previously described (4, 5). Euthanasia by CO_2_ inhalation was performed during week 24 post-diet intervention (at 31 weeks of age). Tissues were snap-frozen in liquid nitrogen and stored at −80°C or placed in 10% neutral buffered formalin fixative for histological analysis.

### Oral Glucose Tolerance Test

Mice were fasted for 6 h (starting at 8 a.m.) and fasted blood glucose was measured using a glucometer (AlphaTRAK 32004-02, Abbott Animal Health) from tail-pricks. Mice were orally administered a filtered aqueous solution (500 mg/mL) of L-dextrose (2 g/kg) and blood glucose levels were measured at 15, 30, 60, 90, and 120-min time-points post-gavage.

### Gastrointestinal Transit Time

During week 10 of diet intervention, GIT was assayed once during the day starting at 9 a.m. and on another day at nighttime starting at 7 p.m. Each mouse was orally administered a solution composed of 1% methyl cellulose, 1.8% NaCl, and 6% carmine red. Mice were placed into empty cages for the duration of the assay with access to food and water. The time required for the first red fecal pellet to be excreted was recorded as gastrointestinal transit time.

### Direct Calorimetry to Determine Caloric Absorption

Fecal calories from fecal samples collected during week 17 of the diet-intervention were determined using a Parr 6772 Precision Thermometer and Parr 6725 Semimicro Calorimeter (Parr Instrument Company, Moline Illinois) Benzoic acid tablets were used as standards. For each mouse, four fecal pellets were immediately collected upon excretion and transferred into a pre-weighed microfuge tube containing sterile deionized water and then weighed again. The weight difference was calculated and recorded as “wet” fecal weight. Fecal samples were then lyophilized (Freeze dryer, Labconco Corporation, Kansas City, Ohio) and compressed into pellets (19–30 mg) for calorimetric measurements. Lyophilized samples were weighed, and the difference between wet and dry fecal samples was recorded as water weight. Fecal caloric density (kcal/g) was calculated using the original wet (undehydrated) fecal weights. Total fecal caloric output was calculated as fecal caloric density (kcal/g) × 28-h fecal output (g). Fecal output was determined by single housing each mouse with fresh bedding and collecting and weighing all feces from bedding after 28 h. Caloric values of LFD, WD, WD-GP diets, and sucrose (kcal/g) were determined by bomb calorimetry. Calories consumed on the day feces were collected for calorimetry were determined as: [caloric density of diet (kcal/g) × intake of food (g)/day) + (caloric density of sucrose (kcal/g) × (intake of 20% sucrose water (mL)/day ^*^ 0.2]. Absorbed calories were calculated as: total kcal consumed—total fecal caloric output. Percent absorptive efficiency was calculated as: kcal absorbed/kcal consumed × 100. Energy efficiency was calculated as: (final body weight—baseline body weight)/(kcal consumed × 123 days).

### Indirect Calorimetry to Determine Metabolic Activity

After 10 and 21 weeks of diet intervention, volume of oxygen consumed (VO_2_), volume of carbon dioxide excreted (VCO_2_), and physical activity (XAMB, YAMB; non-repetitive beam breaks along the x- and y-axis of the cage) were measured using a Comprehensive Laboratory Animal Monitoring System (CLAMS, Columbus Instruments, Columbus, Ohio). Mice were acclimated for 24 h in home cage-style chambers, and measurements collected over the next 48 h were averaged for light (7 a.m.−7 p.m.) and dark (7 p.m.−7 a.m.) cycles. VO_2_, non-protein respiratory exchange ratio (RER), and calorific value (CV) were used to calculate energy expenditure (EE) (kcal/min/kg) based on whole-body weight or lean mass, using the equation VO_2_ × CV = Kcal. CVs were calculated using the equation CV = 3.815 + 1.232 × RER, derived from empirical data from Graham Lusk's “The Elements of the Science of Nutrition (33).”

### Gene Expression

Frozen tissues were homogenized in Qiazol reagent using a Genogrinder (Spex Sample Prep, Metuchen, NJ). RNA was extracted using the RNeasy Plus Universal Mini Kit (QIAGEN^®^, Inc.) and stored at −80°C. RNA (5 μg) was reverse transcribed with random primers (High-Capacity cDNA Reverse Transcription Kit, Applied Biosystems^™^), cDNA was diluted 1:3 and quantitative PCR (qPCR) was performed using TaqMan primers (Life Technologies, Supplementary Table 4). Target mRNA was normalized using endogenous hydroxymethylbilane synthase (*HMBS*) as house-keeping gene for duodenal, jejunal, hepatic, and brown adipose tissue and ribosomal protein, large, P0 (*RPLP0*) for ileal and colonic tissue. Data were analyzed using the 2^−ΔCT^ method.

### *A. muciniphila* qPCR

qPCR of *Akkermansia muciniphila* in fecal samples was performed as previously described (5). Mice were placed into empty cages for immediate collection of fecal pellets upon excretion into microfuge tubes on dry ice. Samples were stored at −80°C. gDNA extracted from fecal samples was diluted to 2.5 ng/μL for quantification of *A. muciniphila* abundance relative to total bacteria and archaea. qPCR was performed using *A. muciniphila* (AM1, AM2) and universal primer (U341F, U515R) sets (5).

### Liver Analyses

Liver sections fixed in 10% neutral buffered formalin were used for hematoxylin and eosin staining (Rutgers Research Pathology Services, Piscataway, NJ) to quantify the area of lipid droplets. Quantification was done on 10 images per liver cross-section at 80× magnification (*n* = 4/group). Images for lipid quantification were captured at multiple locations on each slide that were consistently chosen across samples to prevent selection bias. Quantification was done using Image J1. × (U.S. National Institutes of Health, Bethesda, Maryland; National Center for Microscopy and Imaging Research: ImageJ Mosaic Plug-ins, RRID:SCR_001935) using the “particle analysis” plugin to measure the area of lipid deposits.

### Urinary Glucose

Mice were temporarily placed into a clean cage without bedding and freshly excreted urine was immediately collected from the cage with a filtered pipette tip into a microfuge tube and placed on dry ice followed by storage at −80°C. Urine glucose was measured using a glucometer (AlphaTRAK 32004-02, Abbott Animal Health). Glucometer readings were checked with a standard curve to ensure validity of glucose measurements in water-environments. The standard curve was created using an aqueous solution of L-dextrose diluted to concentrations of 58, 116, 232, 464 mg/dL (Supplementary Figure 2C).

### SCFA and Branched Chain Fatty Acids Quantification

On the day of euthanasia during week 24 of the diet-intervention, colon content was gently pushed from colon segments using a blunt tweezer, snap-frozen, and stored at −80°C. To preserve volatile SCFAs, 1 mL of 0.5 % phosphoric acid in water was added to each weighed sample before allowing to thaw. Short chain fatty acids (i.e., acetate, butyrate, propionate, valerate) and BCFAs (isobutyrate and isovalerate) concentrations were quantified by GC-MS using previously described methods (34).

### L-Lactate Quantification

Duodenum and colon tissues were flushed with cold 1X phosphate buffered saline (pH 7) and cleaned tissues and cecal content were individually placed into microfuge tubes, snap-frozen, and stored at −80°C prior to use. Lactate concentrations were determined using a L-lactate assay kit (Eton Bioscience, San Diego, CA).

### Statistics

Values of *p* < 0.05 were considered significant. Comparison between two groups or time-points were performed using an unpaired two-tailed *t*-test with Welch's correction when sample sizes varied. A comparison between three diet groups was performed by one-way ANOVA followed by Dunnet's multiple comparison test using the WD-group as a control or by Tukey's-test. Different letters and asterisks indicate statistical significance: ^*^p < 0.05, ^**^p < 0.01, ^***^p < 0.001, ^****^p < 0.0001. Correlations were determined using Pearson's correlation analysis. Statistical analysis was performed using Prism 9.0 for Windows (GraphPad Software, La Jolla, CA). Outliers were detected using ROUT's outlier test. Data in all figures are expressed as mean ± standard deviation (SD).

## Results

### GPs Improved Body Composition Despite Greater Caloric Intake

**Figure 1:**
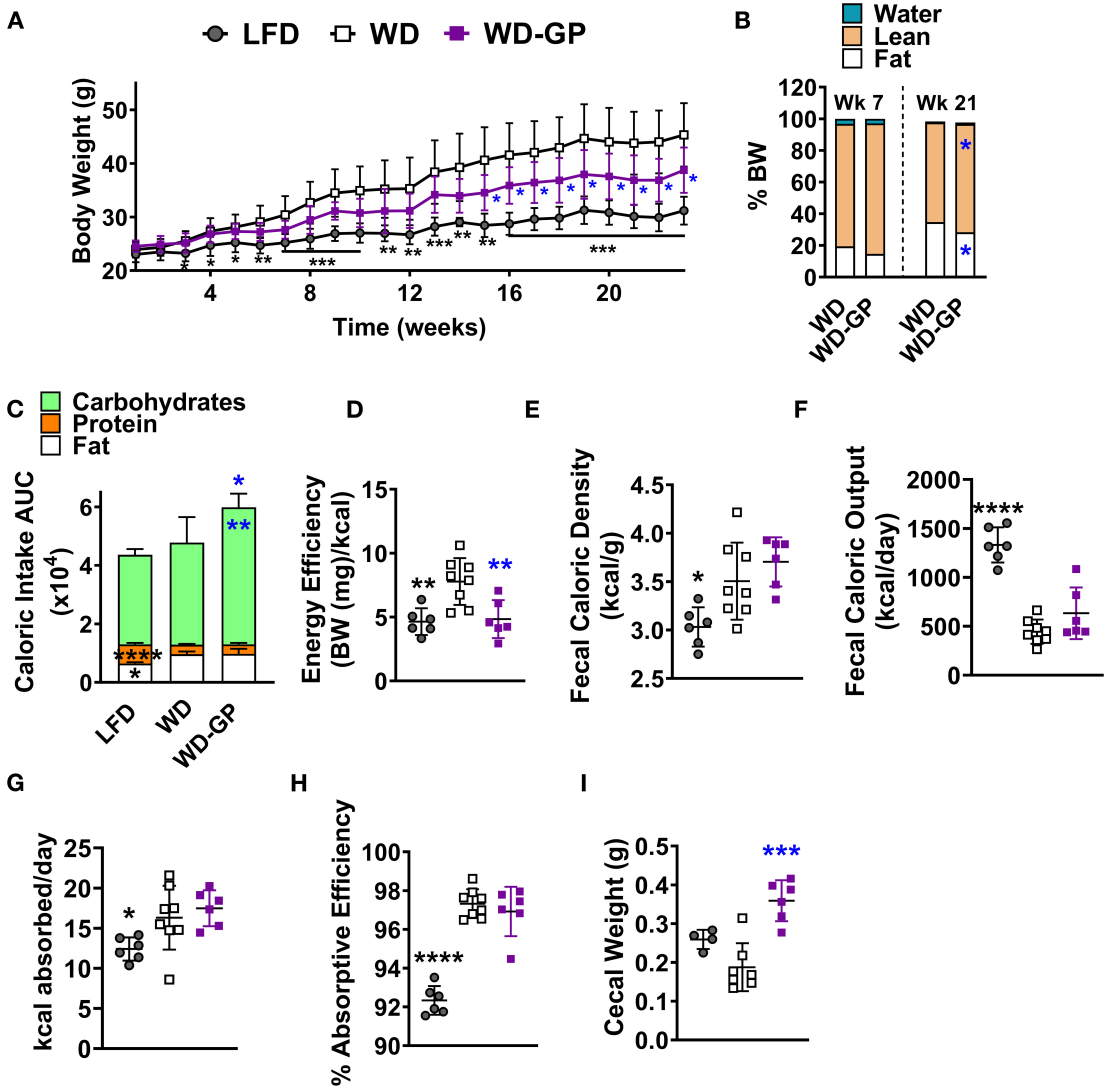
GPs improved body composition and decreased energy efficiency despite greater consumption of calories from carbohydrates. **(A)** Mean weekly body weight for each diet-group (*n* = 6–8). **(B)** Mean body composition, i.e., water, lean, and fat mass as % of body weight (BW), after 7 and 21 weeks of the diet-intervention (*n* = 6–8). **(C)** Mean area under the curve (AUC) of caloric intake from macronutrients from both food and sucrose water over the diet-intervention period (*n* = 6–8/group). Raw data from which AUCs of caloric intake were obtained are shown in Supplementary Figures 2B–E. **(D)** Energy efficiency (mg of body weight gained per kcal consumed) over the diet-intervention. **(E)** Fecal caloric density per gram of feces (wet wt.) and **(F)** total caloric output from feces per day, during week 17 of diet-intervention. **(G)** Calories absorbed per day (calories consumed—calories excreted) and **(H)** absorptive efficiency denotes the percentage of calories absorbed by intestine from calories consumed from food and sucrose water at week 17 of diet-intervention. **(I)** Cecal weight with contents on day of euthanasia (week 24 of diet-intervention). Data are presented as mean ± SD. For **(B)** significant difference between diet groups was determined by an unpaired, two-tailed, *t*-test with Welch's correction and for all other panels significant differences were determined by one-way ANOVA followed by Dunnett's multiple comparisons test with WD group as the control; black colored asterisks indicate statistical significance between the LFD vs. WD group and blue-colored asterisks indicate statistical significance between the WD vs. WD-GP group: ^*^p < 0.05, ^**^p < 0.01, ^***^p < 0.001, ^****^p < 0.0001.

Compared to WD-fed mice, mice fed a WD supplemented with GPs (WD-GP) had significantly lower body weights at several time points from week 15 through 23 (Figure 1A). After 23 weeks, mice in the WD-GP group gained 33% less weight than mice fed a WD (weight increase from baseline for WD: 24.1 ± 5.58 g vs. WD-GP: 15.95 ± 4.48 g, *p* = 0.0086). After 21 weeks of diet intervention, but not 7 weeks, GP-supplemented mice had a lower percentage of fat mass and a higher percentage of lean mass compared to WD-fed mice (Figure 1B). Comparison at individual timepoints showed that mice in WD and WD-GP groups consumed similar amounts of food (Supplementary Figure 1A); however, GP-supplemented mice consumed more calories overall, mainly from carbohydrates (Figure 1C and Supplementary Figures 1B–D) due to a significantly greater average daily intake of sucrose water (WD: 11.44 ± 1.944 mL/day vs. WD-GP: 13.84 ± 0.5 mL/day, *p* < 0.005, Supplementary Figures 1E,F).

An acute test was performed at 11 weeks post-intervention to determine if mice increased sucrose water intake because GPs promoted thirst. Sucrose water was replaced with tap water while food remained available *ad libitum*. When deprived of sucrose water for 2 days, GP-supplemented mice consumed similar amounts of water as WD-fed mice (Supplementary Figure 1G), indicating that GPs did not increase thirst. WD and WD-GP groups increased their food intake when sucrose water was replaced with plain water (Supplementary Figure 1H), likely to replace energy supplied by the sucrose water. Compared to before sucrose water deprivation, WD and WD-GP groups increased sucrose water intake during the first day that sucrose water was returned; however, sucrose water consumption returned to pre-deprivation levels 5 days later (Supplementary Figure 1I).

Despite increased caloric intake, the WD-GP group had less energy efficiency than WD-fed mice, as GP-supplemented mice gained less body weight per calorie consumed over the study period (Figure 1D). These data indicate that GP-supplemented mice could consume more calories than the WD-fed control group yet have lower body weight and adiposity.

### GP Supplementation Did Not Alter Absorption Efficiency

Intestinal absorptive efficiency (calories absorbed/total calories consumed) was evaluated as an explanation for the lower body weight gain in GP-supplemented mice. WD and WD-GP groups had similar fecal caloric densities and total fecal caloric outputs (Figures 1E,F). Because WD and WD-GP groups excreted fewer calories than the LFD group (Figure 1F) they absorbed more calories per day (Figure 1G) and thus had increased absorptive efficiencies (Figure 1H). Compared to WD-fed mice, mice in the WD-GP group had a trend of delayed gastrointestinal transit time at night (WD: 184.6 ± 72.57 min vs. WD-GP: 269.2 ± 99.75, *p* = 0.11), but not during the day when mice were less active (WD: 238.6 ± 120.6 min vs. WD-GP: 299.9 ± 116.4, *p* = 0.32). As in previous studies, GP-supplemented mice had increased cecal weight and size (Figure 1I) (4, 5, 8). Though absorption was unchanged, these intestinal phenotypes suggested that GPs altered nutrient handling along the intestinal tract.

### GP Supplementation Increased Energy Expenditure

**Figure 2:**
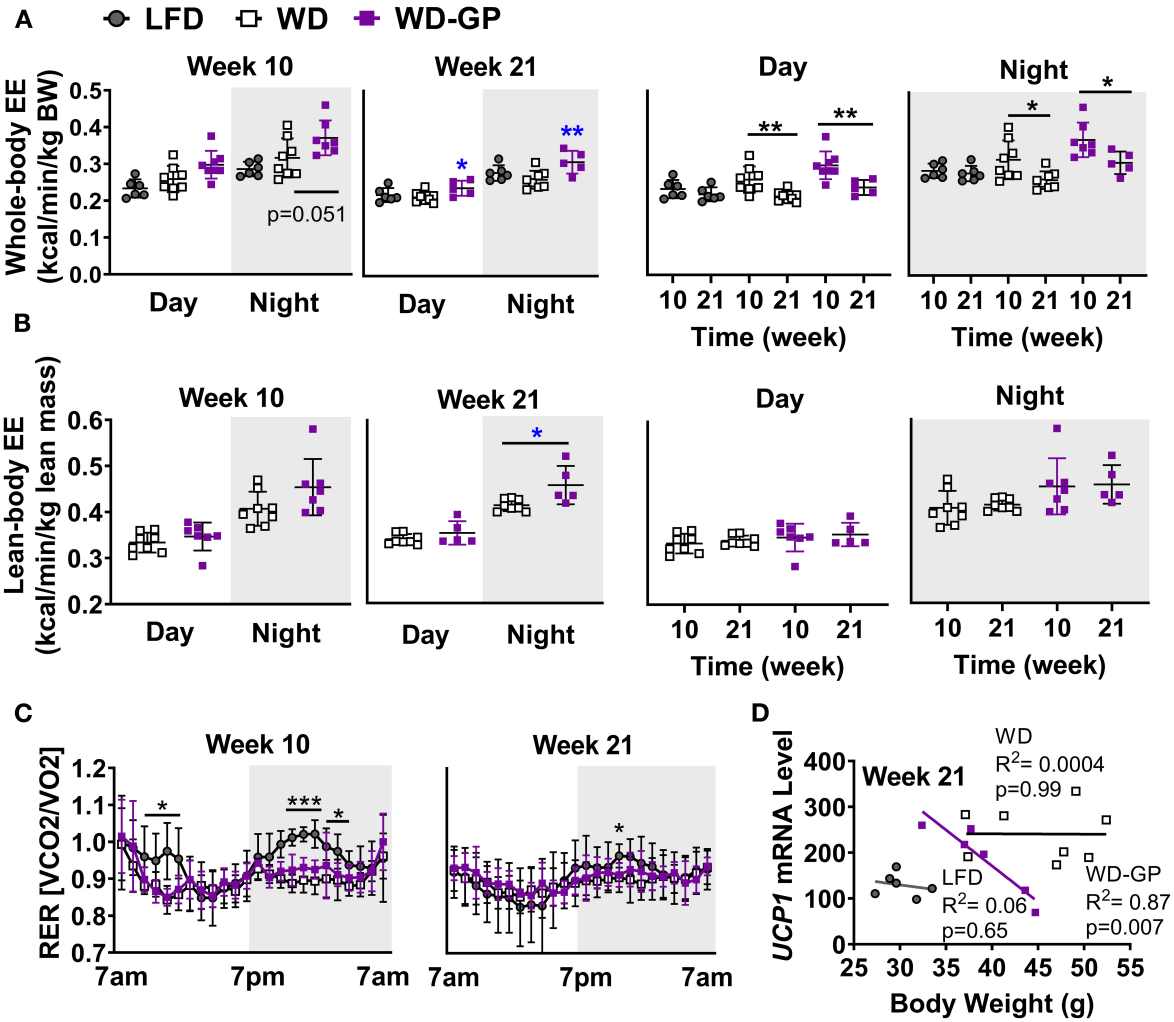
GP supplementation increased energy expenditure. **(A)** Energy expenditure (EE) as kcals expended per minute normalized to kilogram of body weight and **(B)** kilogram of lean mass during day and night phases at week 10 and 21 of the diet-intervention. Left two panels of **A,B** show between group comparisons at weeks 10 and 21, and the right two panels compare weeks 10 and 21 within group. **(C)** Respiratory exchange ratio (RER) at weeks 10 and 21 of diet intervention are shown over time in metabolic chamber. **(D)** Two-tailed Pearson Correlation Analysis between UCP1 mRNA levels in BAT and body weights at week 24. Data are presented as mean ± SD. Significant difference between diet-groups was determined by one-way ANOVA followed by Dunnett's multiple comparisons test using the WD group as control, except in **A** Day and Night graphs and in **B** where an unpaired, two-tailed, *t*-test with Welch's correction was used. Black colored asterisks indicate statistical significance between same diet groups or LFD vs. WD groups while blue-colored asterisks indicate statistical significance between the WD vs. WD-GP group ^*^p < 0.05, ^**^p < 0.01, ^***^p < 0.001. BW, Body weight.

Mice were placed in metabolic chambers during week 10 and 21 of diet intervention to assess EE changes as an explanation for the GP-induced improvement in body composition. Compared to WD-fed mice, the WD-GP group had a trend of higher whole-body EE during the night phase after 10 weeks of diet intervention (Figure 2A). After 21 weeks, whole-body EE in the WD-GP group was higher during day and night periods (Figure 2A). WD and LFD groups had similar levels of whole-body EE (Figure 2A). Within group comparisons showed that at week 21, whole-body EE in WD and WD-GP groups was lower than at week 10, while EE in LFD-fed mice showed no change over this period (Figure 2A). These data suggested that the WD promotes an age-related decline in EE that could not be reversed by GP-supplementation.

Lean mass is inversely related to metabolic syndrome (35), therefore we compared EE normalized to lean body mass, since lean mass was increased by GPs (Figure 1B) and has higher metabolic activity than adipose tissue (36). Lean-body EE of WD-GP-fed mice was only significantly greater than WD-fed mice at week 21 during nighttime (Figure 2B), suggesting that the GP-induced increase in whole-body EE during the day was due to metabolic contributions of multiple tissue compartments and perhaps in part due to increased metabolic activity of lean mass at night. While whole-body EE decreased with time in WD and WD-GP groups, their lean-body EE did not (Figure 2B), indicating that age-related decline of EE was not due to altered metabolic activity of lean tissue.

EE in GP-supplemented mice may be elevated due to increases in fat oxidation, physical activity, or thermogenesis (37). Due to the greater oxygen requirements of fat oxidation, EE is increased, and the respiratory exchange ratio (RER) is reduced. Similar RERs between WD and WD-GP-groups indicate GPs did not alter whole-body metabolism of fat and carbohydrates (Table 1 and Figure 2C). At week 10 during the night phase, LFD-fed mice had a significantly higher RER than WD and WD-GP groups (Table 1 and Figure 2C) indicating increased carbohydrate over fat metabolism, but by week 21 this difference was lost suggesting an age-related decline in RER of LFD-fed mice (Figure 2C). GP supplementation did not affect physical activity (Table 1) therefore increased movement could not explain the increased EE. Peroxisome proliferative activated receptor-gamma, coactivator 1 (PCG1α) encoded by *Ppargc1a* is a transcriptional coactivator regulating mitochondrial biogenesis. PCG1α also induces non-shivering thermogenesis by promoting transcription of uncoupling protein 1 (UCP1) in brown adipose tissue (BAT). Compared to WD-fed mice, GP-supplementation did not significantly affect gene expression of *Ucp1* or *Ppargc1a* in BAT (Supplementary Table 5). While gene expression of *UCP1* was not upregulated in GP-supplemented mice, a significant inverse relationship between body weight and *UCP1* mRNA levels was observed that was absent in WD- and LFD-fed mice (Figure 2D). GP-supplementation may increase the thermogenic contributions of *UCP1*.

**Table 1:** Metabolic activity measured by indirect calorimetry.

		**LFD**			**WD**	**WD-GP**		
	**Week 10**							
Day	VCO_2_ (mL/kg BW/h)	2,657 ± 362.5	0.69	ns	2,809 ± 150.4	3,234 ± 122.9	0.07	ns
	VO_2_ (mL/kg BW/h)	2,865 ± 268.0	0.32	ns	3,160 ± 152.5	3,638 ± 161.7	0.053	ns
	RER	0.87 ± 0.08	0.74	ns	0.889 ± 0.012	0.892 ± 0.011	0.91	ns
	EE (mL/kg BW/h)	0.233 ± 0.02	0.31	ns	0.259 ± 0.012	0.298 ± 0.013	0.06	ns
	Movement (XAMB^*^YAMB)	34,379 ± 18,652	0.75	ns	25,681 ± 27,850	21,993 ± 20,744	0.93	ns
Night	VCO_2_ (mL/kg BW/h)	3,362 ± 245.1	0.88	ns	3,483 ± 213.1	4,098 ± 191.9	0.05	^*^
	VO_2_ (mL/kg BW/h)	3,430 ± 240.8	0.28	ns	3,848 ± 225.7	4,419 ± 203.3	0.08	ns
	RER	0.97 ± 0.03	0.0004	^***^	0.905 ± 0.007	0.926 ± 0.011	0.24	ns
	EE (mL/kg BW/h)	0.29 ± 0.02	0.37	ns	0.316 ± 0.019	0.371 ± 0.018	0.051	ns
	Movement (XAMB^*^YAMB)	199,272 ± 135,011	0.38	ns	130,820 ± 117,993	68,616 ± 39,712	0.39	ns
	**Week 21**							
Day	VCO_2_ (mL/kg BW/h)	2,356 ± 331.8	0.99	ns	2,312 ± 81.06	2,383 ± 220.4	0.92	ns
	VO_2_ (mL/kg BW/h)	2,689 ± 200.0	0.70	ns	2,611 ± 55.21	2,904 ± 110.1	0.03	^*^
	RER	0.87 ± 0.09	0.90	ns	0.883 ± 0.017	0.889 ± 0.01	0.78	ns
	EE (mL/kg BW/h)	0.22 ± 0.02	0.79	ns	0.213 ± 0.005	0.238 ± 0.009	0.05	^*^
	Movement (XAMB^*^YAMB)	31,879 ± 30,907	0.04	^*^	7,468 ± 4,110	10,463 ± 3,302	0.94	ns
Night	VCO_2_ (mL/kg BW/h)	3,138 ± 201.1	0.26	ns	2,876 ± 127.7	3,402 ± 165	0.02	^*^
	VO_2_ (mL/kg BW/h)	3,374 ± 271.6	0.39	ns	3,174 ± 93.91	3,737 ± 168	0.008	^**^
	RER	0.93 ± 0.023	0.32	ns	0.903 ± 0.016	0.913 ± 0.012	0.86	ns
	EE (mL/kg BW/h)	0.28 ± 0.02	0.34	ns	0.261 ± 0.009	0.308 ± 0.014	0.008	^**^
	Movement (XAMB^*^YAMB)	189,253 ± 134,473	0.01	^*^	48,130 ± 38,088	91,988 ± 63,058	0.59	ns

*Data are expressed as mean ± SD along with statistical significance compared to the WD-group determined by a one-way ANOVA followed by Dunnett's multiple comparison test. ns, p > 0.05*,

* *p < 0.05*,

** *p < 0.01*,

*** *p < 0.001*.

BW, Body Weight; RER, Respiratory Exchange Ratio; XAMB^*^YAMB, Ambulatory movements along x-axis^*^y-axis.

### Oral Glucose Tolerance and Intestinal Markers of Insulin Release Were Similar Across Diet Groups

Mice in the WD and WD-GP groups had similar OGT to LFD-fed mice after 3, 12, and 23 weeks (Supplementary Figure 2A). Urinary glucose was assayed to evaluate kidney function, as renal glucose reabsorption contributes to the maintenance of euglycemia (38). The WD and WD-GP groups had similar urinary glucose concentrations while that of LFD-fed mice was increased (Supplementary Figures 2B,C). Prohormone convertase 1 (*Pcsk1*) cleaves preproglucagon protein (GCG) to produce GLP-1, an incretin peptide stimulating insulin release. *Gcg* and *Pcsk1* gene expression in duodenal and colon tissue was unaltered by GP-supplementation (Supplementary Table 5).

### GP Supplementation Reduced WD-Induced Hepatic Steatosis

**Figure 3:**
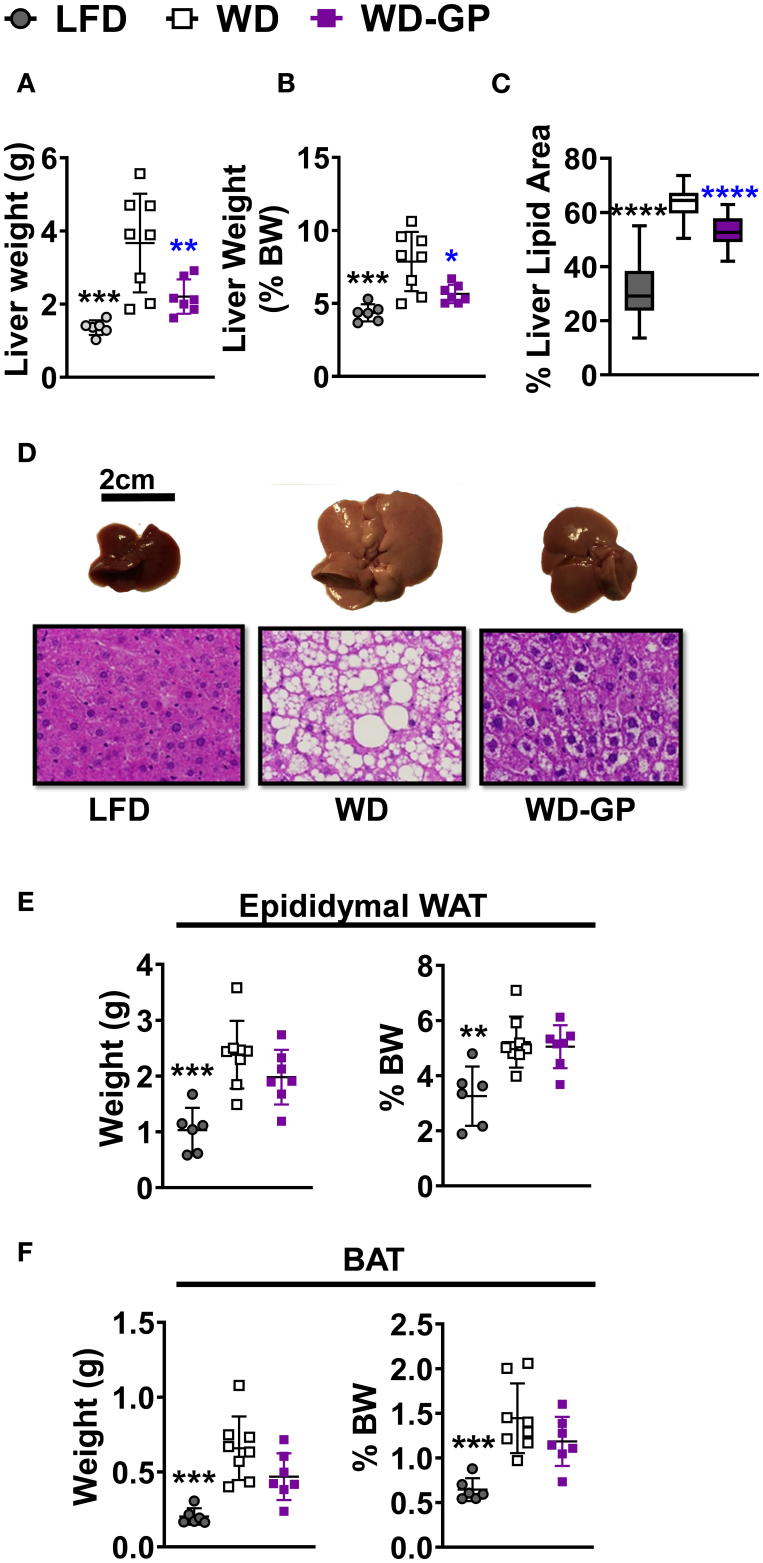
GP supplementation diminished WD-induced hepatic steatosis. **(A)** Liver weights at endpoint, after 23 weeks of diet-intervention, **(B)** Liver weight as a percentage of body weight. **(C)** Mean percentage of liver coverage by lipid droplets per cross-section, as quantified from hematoxylin and eosin (H&E) staining (*n* = 3/group); **(D)** Representative images of murine livers from each diet group with corresponding H&E-stained liver sections (below) showing white lipid droplets; images were captured at 40× magnification. Organ weights as raw weight (left panel) and as percent of body weight (right panel) of **(E)** white adipose tissue (WAT), and **(F)** brown adipose tissue (BAT) on day of euthanasia (week 24 of diet-intervention). Data are presented as mean ± SD. Significant differences between groups were determined by one-way ANOVA followed by Dunnett's multiple comparisons test with the WD group as control. Black colored asterisks indicate statistical significance between the LFD vs. WD group and blue-colored asterisks indicate statistical significance between the WD vs. WD-GP group. ^*^p < 0.05, ^**^p < 0.01, ^***^p < 0.001, ^****^p < 0.0001. BW, Body weight.

GP supplementation reduced the WD-induced increase in hepatic weight (Figures 3A,B) and hepatic adiposity (Figure 3C), as revealed by reduced lipid droplet area and nuclei displacement (Figure 3D). Compared to WD-fed mice, GP supplementation did not reduce the percentage of body weight contributed by white adipose tissue (WAT) and BAT (Figures 3E,F). Increased expression of hepatic fibroblast growth factor-21 (*FGF21*), which regulates adiposity, insulin sensitivity, and carbohydrate intake, has been associated with NAFLD (39). GP-supplementation did not alter *FGF21* mRNA levels (Supplementary Table 5). To investigate whether hepatic steatosis may be due to altered lipid metabolism, expression of lipogenic enzyme fatty acid synthase (*FAS*) and lipolytic enzyme carnitine palmitoyl-transferase (*CPT1a*) was measured. GP supplementation did not alter *FAS* or *CPT1a* mRNA levels (Supplementary Table 5); however, substrate concentrations can be the main driver of lipogenic pathways rather than transcription (40).

### GPs Altered the Colonic SCFA Profile

**Figure 4:**
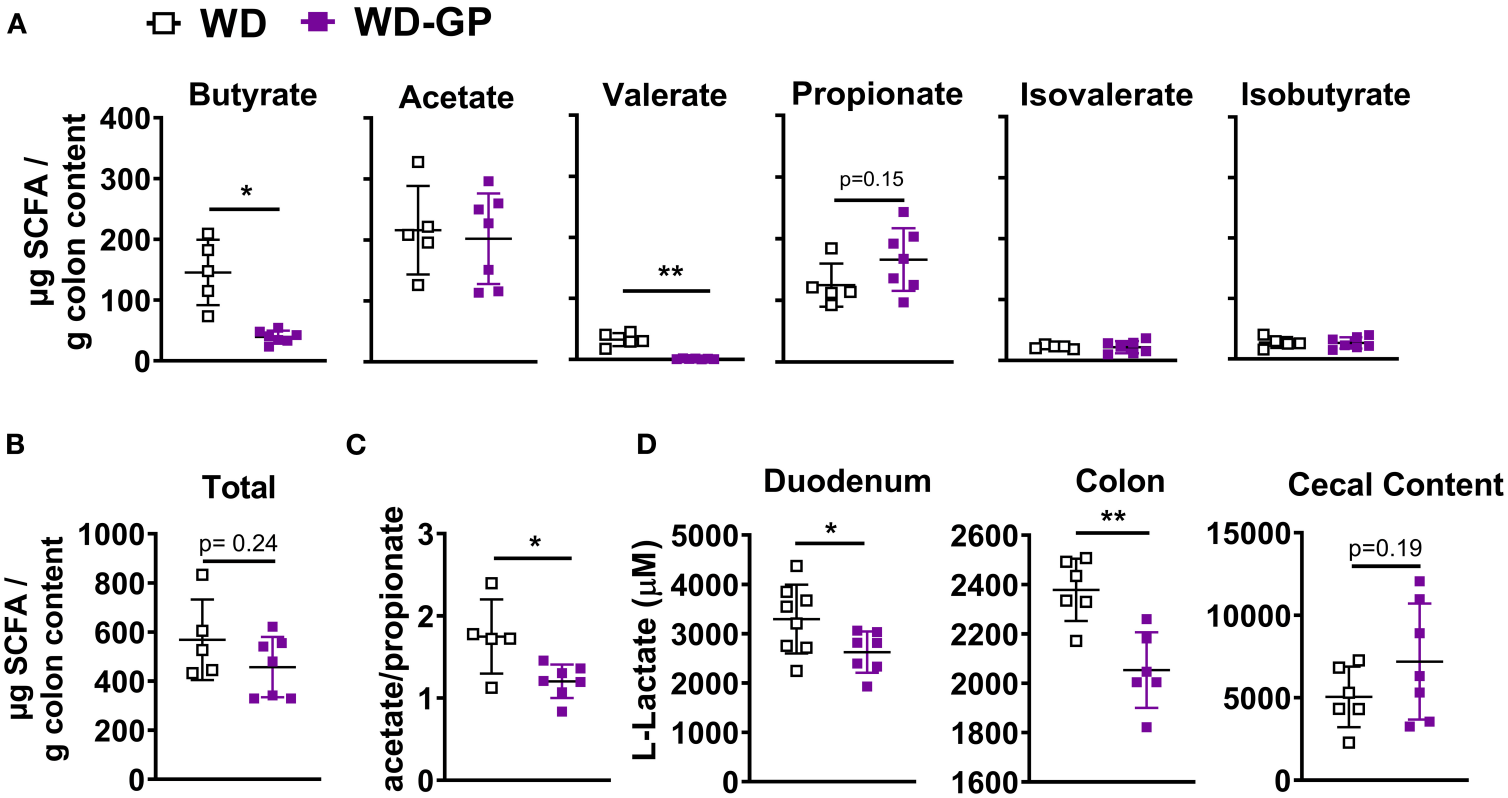
GP supplementation decreased intestinal concentrations of specific SCFAs and lactate. **(A)** Concentrations of individual SCFAs and BCFAs in colon content. **(B)** Total SCFA and BCFA concentration. **(C)** Ratio of acetate to propionate. **(D)** Lactate concentrations in duodenum and colon tissues and in cecal content. Data are presented as mean ± SD. Significant difference was determined by unpaired, two-tailed, *t*-test with Welch's correction; ^*^p < 0.05, ^**^p < 0.01.

We hypothesized that GPs may improve metabolic functions by reducing concentrations of lipogenic SCFAs. Butyrate provides 60–70% of energy requirements to colonocytes (14) and is the preferred energy source of ileal cells (15). Remaining SCFA are excreted, converted to glucose, or sent to the liver *via* enterohepatic circulation and incorporated into lipids, providing 5–10% of whole-body energy needs (14, 22, 40). Butyrate and acetate are major substrates for *de novo* lipogenesis in colonocytes (41) and hepatic cells (42). In contrast, propionate is a gluconeogenic precursor in enterocytes (42) and hepatic cells (21, 43). Increased levels of SCFAs are associated with diet induced-NAFLD and obesity (7, 23–29), therefore SCFA concentrations were measured in colon content. Compared to WD-fed mice, the WD-GP group had reduced concentrations of butyrate and valerate in colon content while acetate, propionate, isovalerate, and isobutyrate levels were similar (Figure 4A). WD and WD-GP groups had similar concentrations of total SCFAs (Figure 4B). Compared to the WD group, GP-supplemented mice had a reduced ratio of acetate:propionate (Figure 4C). Propionate has been shown to compete with acetate for acetyl-coA synthetase in liver mitochondria and inhibit incorporation of acetate into lipid synthesis, therefore, a reduced acetate:propionate is a marker for reduced hepatic lipogenic contributions from SCFAs (21, 43). Additionally, reductions to putrefactive valerate may be beneficial, as high levels of this protein fermentation product are detrimental to gut health (19).

**Figure 5:**
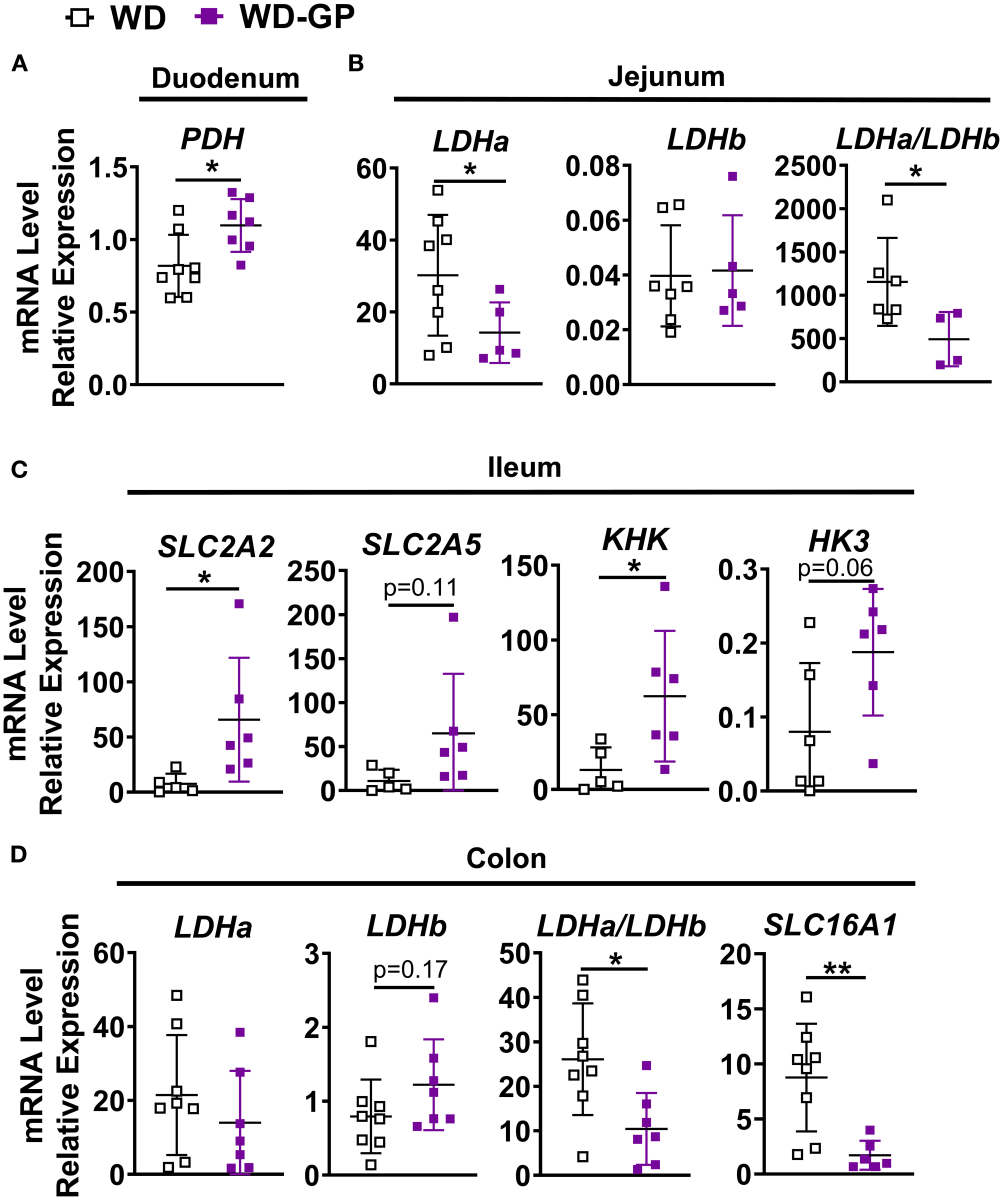
Intestinal gene expression. qPCR analyses showing relative mRNA levels of indicated genes or gene ratios expressed in **(A)** duodenum **(B)** jejunum, **(C)** ileum and **(D)** colon tissues of mice fed WD or WD-GP (*n* = 6–8 mice per group). Data represent technical duplicates analyzed by 2^−ΔCT^ method. Data are presented as mean ± SD. Significant difference was determined by an unpaired, two-tailed, *t*-test with Welch's correction; ^*^p < 0.05, ^**^p < 0.01.

To determine if butyrate and valerate levels in colon digesta of GP-supplemented mice were reduced due to increased host-absorption, we measured duodenal, jejunal, ileal, colonic and hepatic mRNA levels of monocarboxylase transporter 1 (MCT-1, encoded by *SLC16A1*), which transports both lactate and SCFAs into cells. *SLC16A1* was decreased in colon tissue of GP-supplemented mice but was unchanged in the small intestine and liver (Figure 5D and Supplementary Table 5). *In vitro* treatment of human fecal microbiota with PACs reduced microbial SCFA production (9), indicating that the decreased butyrate and valerate levels observed in colon content was likely due to decreased bacterial production of these SCFAs rather than increased host-absorption.

A bloom in intestinal *A. muciniphila* is a reproducible effect of GP supplementation (4, 5, 8). GP-supplementation promoted a bloom in *A. muciniphila* (WD: 14.34% vs. WD-GP: 34.12% relative abundance; *p* < 0.0001) while total bacterial and archaea remained similar between WD and WD-GP-fed mice (Supplementary Figures 3A,B), as previously reported (4, 5). *Akkermansia muciniphila* produces propionate as a result of metabolizing fermentable fiber or host intestinal mucins (44); however, an inverse relation between *A. muciniphila* relative abundance and fecal SCFA concentrations was reported (23). GP supplementation did not significantly change propionate or total SCFA concentrations (Figure 4). As SCFAs are a significant energy source for the gut, we next evaluated the effects of altered SCFA profile on intestinal metabolism.

### GPs Upregulate Intestinal Metabolism of Carbohydrates

Butyrate is largely oxidized by the ileum and colon for energy (14, 15); therefore, we hypothesized that the GP-induced reduction in butyrate would trigger intestinal epithelial cells to metabolize other energy substrates. Indeed, multiple molecular changes noted within intestines of GP-treated mice supported a shift to carbohydrate metabolism.

Expression of genes for glucose and fructose transporters including sodium-glucose transporter 1 (SGLT1*; SLC5A1*), glucose transporter-2 (GLUT2; *SLC2A2*) and GLUT5 (*SLC2A5*) were analyzed in duodenum, jejunum, ileum, and colon tissue. mRNA levels of these transporters were unchanged in all tissue (Supplementary Table 5), except for in the ileum where *SLC2A2*, an apical and basolateral glucose and fructose transporter (45), was significantly increased by GP-supplementation (Figure 5C). Enzymes hexokinase (*HK3*) and ketohexokinase (*KHK*) phosphorylate glucose and fructose upon entrance into cells to prepare for glycolytic and fructolytic pathways. Compared to WD-fed mice, ileal tissues of mice fed WD-GP showed increased mRNA levels of *KHK* and a trend of increased *HK3* (p = 0.063), suggesting greater glucose and fructose utilization (Figure 5C). Glucose-6-phosphatase (G6Pase; *G6Pc*) mRNA levels were similar in liver, duodenum, jejunum, and ileum tissues (Supplementary Table 5) indicating no significant difference in gluconeogenesis or glucose release into circulation.

Pyruvate dehydrogenase complex (PDH) is a multi-subunit enzyme within the mitochondrial matrix that catalyzes the conversion of pyruvate to acetyl-coA during oxidative glucose metabolism. Compared to WD-fed mice, GP-supplemented mice had increased duodenal PDH complex component X (*PDHx*) mRNA, which is indicative of increased oxidative capacity (Figure 5A). WD and WD-GP had similar *PDHx* mRNA levels in jejunum and ileum (Supplementary Table 5).

Lactate dehydrogenase (LDH) subunit a catalyzes the interconversion of pyruvate to lactate and LDH subunit b catalyzes the opposite reaction, therefore the ratio of *Ldha/Ldhb* is used to determine the dominant direction of pyruvate-to-lactate interconversion. GP-supplemented mice had a decreased *Ldha/Ldhb* gene transcript ratio in jejunal and colon tissue (Figures 5B,D) and reduced L-lactate concentrations within duodenal and colon tissues (Figure 4D), suggesting reduced anaerobic respiration. WD and WD-GP groups had similar L-lactate concentrations in cecal content (Figure 4D), indicating that reduced lactate levels in tissue was not simply due to reduced microbial lactate production. Overall, these data suggest intestinal oxidation of glucose and fructose was increased in GP-supplemented mice.

## Discussion

In this study we investigated multiple mechanisms by which GPs could mitigate WD-induced disruptions to energy balance. *In vitro* studies have shown that PAC-rich polyphenol extracts can inhibit digestive enzymes required for intestinal nutrient absorption (46, 47); however, GP supplementation did not affect absorptive efficiency in WD-fed mice. Rather, reduced weight gain in GP-supplemented mice was associated with increased EE. HFD-induced mitochondrial dysfunction leads to increased anaerobic glycolysis (48), decreased EE (49), and contributes to NAFLD and cardiometabolic disease (50–52). Antioxidant polyphenols protect mitochondrial functions (53), which likely attenuate the HFD- or WD-induced decline in EE. GPs decreased HFD-induced accumulation of reactive oxidative species in the gut (6) and thus may protect intestinal mitochondrial oxidative capacity. Similar to GP-supplementation, mice fed a HFHS-diet supplemented with a PAC-rich camu camu extract had increased EE in association with a bloom in *A. muciniphila* (32). Treatment of diet-induced obese mice with pasteurized *A. muciniphila* increased whole-body EE (54), suggesting that the observed GP-associated increase in EE is directly related to *A. muciniphila*. Thus, reducing intestinal oxidative stress and increasing *A. muciniphila* are two mechanisms by which GPs may improve energy balance. Additional studies are needed to determine whether there is a causal relationship between GP-induced changes in SCFA concentrations and EE.

**Figure 6:**
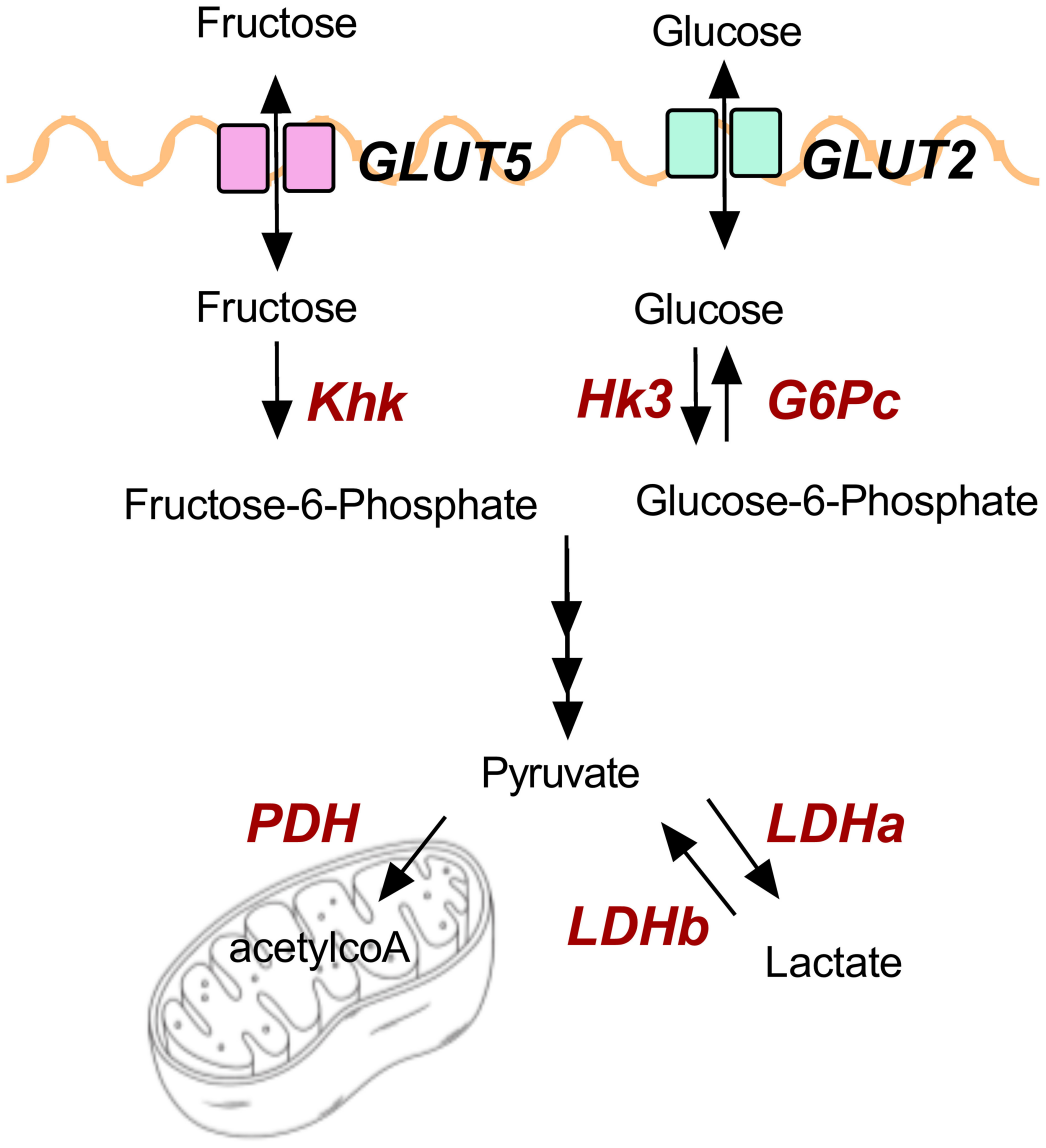
Illustration of genes and products involved in carbohydrate metabolism.

GP-supplemented mice had less hepatic steatosis despite increased sucrose water consumption. Recent findings showed that the small intestine can convert 90% of fructose to glucose to shield the liver from toxic fructose exposure, though excess fructose can reach the colonic microbiota and be metabolized into SCFAs (55). Fructose and fecal SCFAs have been linked to NAFLD (29, 56). GP-supplemented mice consumed more fructose *via* sucrose water, but OGT was not altered supporting the idea that carbohydrates were being metabolized by the intestine rather than being shuttled to the liver. Indeed, increased intestinal transcription of markers for carbohydrate uptake, metabolism, and oxidation suggest GP supplementation increased carbohydrate utilization. Figure 6 illustrates glucose and fructose transporters (GLUT2, GLUT5) and the enzymes (in red text) that were found to be altered by GP supplementation.

Butyrate concentrations measured in colon content of WD and WD-GP groups of mice (23.5–209 μg/mg of colon content) were comparable to other mouse studies reporting a mean of ~112 μg/mg (57). GPs reduced concentration of butyrate almost 4-fold on WD. As GP supplementation promoted depletion of butyrate, a major fuel source for ileal and colon cells (14, 15), we hypothesized that this triggered intestinal cells to seek carbohydrates as a fuel source. Furthermore, butyrate has been shown to inhibit activity of the PDH complex (58), which was consistent with the inverse relationship between butyrate levels and carbohydrate oxidation observed in GP-supplemented mice. Less portal vein transport of fructose and butyrate and the decreased acetate:propionate ratio together likely contributes to reduced hepatic fat accumulation observed in GP-supplemented mice. Future studies measuring fructose, glucose and SCFAs in different milieus (i.e., ileal and cecal content, portal blood and circulation) would be useful to determine if a GP-induced reduction in SCFAs and increased intestinal carbohydrate metabolism can together contribute to reduced delivery of lipogenic substrates to the liver.

Lower butyrate levels indicate decreased microbial-energy harvest in mice fed WD-GP, which may explain similarities with antibiotic-treated mice that receive less energy substrate from the gut microbiota.

Similar to GP-treated mice, antibiotic treated mice showed weight loss and a bloom in *A. muciniphila* (59). We previously noted reduced α-diversity but unchanged total bacterial load in the gut microbiome of GP-treated mice, suggesting GPs may exert an antimicrobial effect on certain microbial members while providing an expanded niche for *A. muciniphila* (4, 8). Similar to GP-supplemented mice, diminished butyrate levels in antibiotic-treated mice was associated with increased transcription of glycolytic enzymes such as hexokinase, indicating increased carbohydrate metabolism (60). These studies suggest that when gut microbial metabolism is reduced, the proximal intestine compensates by absorbing and metabolizing more dietary carbohydrates.

Interestingly, the antidiabetic drug metformin or Roux-en-Y gastric bypass (RYGB), are associated with increased abundance of *A. muciniphila* (61, 62), upregulated carbohydrate metabolism (63–65), reduced SCFA levels (66, 67), lower intestinal lactate concentrations (68), and an elevated EE independent of UCP1 protein levels (68). Increased carbohydrate metabolism from RYGB was said to be triggered by Roux limb exposure to undigested nutrients in the small intestine (64). Increased gastrointestinal transit time and cecal size in GP-supplemented mice also suggests increased contact between the small intestine and luminal contents. While these parallels may be coincidental, they also suggest common mechanisms across these interventions.

In this study the WD, delivering 46% kcal fat from mainly butter and 35% kcal carbohydrate from mainly sucrose, caused fatty liver independent of hyperglycemia. Previously, mice fed a HFD containing 62% kcal fat from lard, and 7.6% kcals from sucrose, had worse OGT than LFD-fed mice while mice fed HFD supplemented with GPs showed better OGT than HFD-fed controls (4, 5, 69). Energy provided by the HFD (21 kJ/g) used in prior studies (4, 5, 69) and the WD (20 kJ/g) used in this study were comparable. We considered that the lower fat concentration of the WD protected mice from glucose intolerance; however, this may not be an adequate explanation. Mice fed a HFHS diet with a similar macronutrient ratio (45% kcals from lard fat and 35% carbohydrate mainly from sucrose) developed glucose intolerance compared to LFD-fed mice after just 7 weeks (70). Alternatively, the fatty acid composition of butter used in the WD formulation may be less disruptive to glucose homeostasis than lard as a meta-analysis showed a positive correlation between moderate butter intake and reduced type 2 diabetes-incidence (71). Supplementary Table 3 shows the fatty acid composition of lard vs. butter, which contains butyric acid. Mice in the WD and WD-GP-groups consumed on average 0.3–0.4 g butyric acid /kg BW per day. Oral supplementation of butyrate has been reported to be protective against both NAFLD (72) and insulin resistance (73), yet increased levels of butyrate-producing bacteria and levels in feces has also been linked to these diseases (23–25, 27–29). Providing dietary butyric acid in butterfat may have protected mice fed WD and WD-GP against hyperglycemia. The studies suggest that butyrate may have differential affects in the context of different diets and delivery to the proximal intestine *via* dietary consumption vs. to the distal intestine *via* microbial production. Studies which compare effects of butyrate in the proximal verses distal gut on obesogenic diets are required to answer these questions.

In summary, GP-induced changes to the SCFA profile may contribute to metabolic resilience by increasing intestinal carbohydrate oxidation and reducing hepatic delivery of lipogenic carbohydrates and butyrate while the bloom in *A. muciniphila* may be responsible for the improved EE. These data provide evidence for the intestinal milieu's involvement in GP-mediated health benefits as well as insight into the roles of SCFAs in obesogenic conditions. Further understanding GP bioactivity within the intestine will contribute to evidence-based dietary recommendations and clinical applications.

## Data Availability Statement

The raw data supporting the conclusions of this article will be made available by the authors, without undue reservation.

## Ethics Statement

The animal study was reviewed and approved by Rutgers University Institutional Care and Use Committee.

## Author Contributions

EM and DER planned and designed experiments. EM, KAK, QH, KS, KMT, and RMD contributed to animal care, metabolic phenotyping, sample collection, and molecular assays. KAK quantified lipid droplets from hepatic cross-sections. QH helped organize metabolic chamber data. EM analyzed all data and drafted the manuscript. DER provided oversight for the work and edited the final manuscript. All authors read and approved the final manuscript.

## Conflict of Interest

DER has equity in Nutrasorb LLC. The funders had no role in the design of the study; in the collection, analyses, or interpretation of data; in the writing of the manuscript, or in the decision to publish the results. The remaining authors declare that the research was conducted in the absence of any commercial or financial relationships that could be construed as a potential conflict of interest.

## Abbreviations

EE: Energy expenditure
GP: grape polyphenol
HFD: high fat diet
HFHS: high fat high sucrose
LFD: low fat diet
NAFLD: non-alcoholic fatty liver disease
OGT(T): oral glucose tolerance (test)
PAC: proanthocyanidin
SCFA: short chain fatty acid
WD: Western diet
WD-GP: Western diet with grape polyphenols.
