# Supplementary material for: Grape Polyphenols Attenuate Diet-Induced Obesity and Hepatic Steatosis in Mice in Association With Reduced Butyrate and Increased Markers of Intestinal Carbohydrate Oxidation
Source: Front Nutr. 2021 Jun 14;8:675267. doi: 10.3389/fnut.2021.675267 (PMC8238044; doi:10.3389/fnut.2021.675267)
Supplement: Supplementary file 1 [file Data_Sheet_1.pdf]

Supplementary Material

1 Supplementary Tables

Supplementary Table 1. Nutritional composition of grape polyphenol-soy protein isolate (GP-SPI) complex and SPI (per 100 g)

Diet	SPI	GP-SPI
Calories	386	392
Calories from fat	29	40
Ash (%)	3.9	4.5
Moisture (%)	4.6	3.0
Carbohydrates (%)	0	31.3
Total Dietary Fiber	< 0.1	21.0
Protein (%)	89.3	56.7
Total Fat (%)	3.24	4.4
Sugars (%)		
Glucose	< 0.1	1.15
Fructose	< 0.1	1.58
Sucrose	< 0.1	0.35
Total Sugars (%)	0	3.08

Supplementary Table 2. Mouse diet formulations

	LFD	WD	WD-GP
**Ingredient (g)**			
Casein	75.5	95.5	95.5
Soy Protein, Supro 661	0	0	36.3
DL – Methionine	3	3	3
GP-SPI	0	0	96.8
Soy Protein	116.5	96.8	0
Corn Starch	641.9	0	0
Maltodextrin 10	150	99.1	91.6
Sucrose	0.4	340.3	340
Dextrose	1.1	0.9	0
Fructose	1.6	1.3	0
Cellulose	50	50	29.7
Butter	42.5	200	200
Corn Oil	30	30	27.3
Cholesterol	0	1.5	1.5
Ethoxyquin	0.04	0.04	0.04
Mineral Mix S10001	35	35	35
Calcium Carbonate	4	4	4
Vitamin Mix V10001	10	10	10
Choline Bitartrate	2	2	2
Red Dye #40, FD&C	0	0	0.025
Blue Dye #1, FD&C	0.05	0.025	0
Yellow Dye #5, FD&C	0	0.025	0.025
**Total (g)**	1163.59	969.49	972.79
**Kcal%**			
Protein	15	15	15
Carbohydrate	70	39	39
Dextrose	0.1	0.1	0.1
Sucrose	1	30	30
Fructose	0.1	0.1	0.1
Fat	15	46	46
**Total**	100	100	100
**Kcal/gm**	3.96	4.75	4.73
**kJ/gm**	16.58	19.90	19.84

Supplementary Table 3. Composition of dairy butter based used in LFD, WD, and WD-GP-diets, and of lard used in our previous studies, based on manufacturer data

Ingredient	Butter, Anhydrous<br>gm	Lard
C2, Acetic	0.0	0.0
C4, Butyric	3.2	0.0
C6, Caproic	1.9	0.0
C8, Caprylic	1.1	0.0
C10, Capric	2.5	0.1
C12, Lauric	2.8	0.1
C14, Myristic	10.0	1.2
C14:1, Myristoleic	1.5	0.0
C15	N/A	0.1
C16, Palmitic	N/A	20.0
C16:1, Palmitoleic	2.3	1.5
C17	N/A	0.3
C18, Stearic	12.1	11.0
C18:1, Oleic	25.1	34.9
C18:2, Linoleic	2.3	21.9
C18:3, Linolenic	1.4	1.3
C18:4, Stearidonic	0.0	0.0
C20, Arachidic	1.0	0.2
C20:1	0.0	0.6
C20:2	0.0	0.8
C20:3	0.0	0.1
C20:4, Arachidonic	0.0	0.3
C20:5, Eicosapentaenoic	0.0	0.0
C21:5	0.0	0.0
C22, Behenic	0.0	0.0
C22:1, Erucic	0.0	0.0
C22:4, Clupanodonic	0.0	0.0
C22:5, Docosapentaenoic	0.0	0.1
C22:6, Docosahexaenoic	0.0	0.0
C24, Lignoceric	0.0	0.0
C24:1	0.0	0.0
**Total**	93.4	94.3
Saturated (g)	60.8	32.9
Monounsaturated (g)	28.9	37.0
Polyunsaturated (g)	3.7	24.4
**Saturated (%)**	65.1	34.9
**Monounsaturated (%)**	31.0	39.2
**Polyunsaturated (%)**	4.0	25.9

Supplementary Table 4. Taqman assays

TaqMan primer	Assay ID

CPT1a	Mm01231183_m1
FAS	Mm01204974_m1
FGF21	Mm07297622_g1
G6PC	Mm00839363_m1
Gcg	Mm00801714_m1
HK3	Mm01341942_m1
HMBS	Mm01143545_m1
KHK	Mm00434647_m1
LDHa	Mm01612132_g1
LDHb	Mm05726463_g1
Pcsk1	Mm00479023_m1
Ppargc1a	Mm01208835_m1
SLC16A1	Mm01306379_m1
PDHx	Mm00558275_m1
RPLP0	Mm00725448_s1
SLC5A1	Mm00451203_m1
SLC2A2	Mm00446229_m1
SLC2A5	Mm00600311_m1
UCP1	Mm01244861_m1

Supplementary Table 5. Gene Expression Data

Tissue	Gene	WD-GP (n= 5 – 8)	WD (n= 6 – 8)	p-value	Sig.
Liver	*CPT1a*	5.79 ± 0.93	6.67 ± 1.87	0.30	ns
	FAS	0.17 ± 0.02	0.2 ± 0.07	0.29	ns
	FGF21	0.40 ± 0.035	0.37 ± 0.40	0.80	ns
	G6PC	3.35 ± 2.42	3.13 ± 1.99	0.87	ns
	HK3	0.02 ± 0.01	0.02 ± 0.006	0.96	ns
	KHK	7.18 ± 1.11	10.95 ± 5.32	0.11	ns
	SLC16A1; MCT-1	1.27 ± 0.66	1.09 ± 0.2	0.55	ns
	SLC2A2; GLUT2	4.98 ± 2.02	5.00 ± 0.59	0.98	ns
	SLC2A5; GLUT5	0.31 ± 0.16	0.39 ± 0.17	0.40	ns
Brown adipose	*UCP1*	185.6 ± 76.45	240.7 ± 58.75	0.18	ns
	Ppargc1a	0.75 ± 0.22	0.57 ± 0.09	0.11	ns
Duodenum	*G6PC*	5.3 ± 2.01	9.04 ± 4.87	0.09	ns
	Gcg	0.65 ± 0.19	0.72 ± 0.25	0.57	ns
	HK3	0.023 ± 0.01	0.019 ± 0.006	0.48	ns
	KHK	19.19 ± 5.96	23.67 ± 10.26	0.35	ns
	LDHa	23.72 ± 3.83	24.07 ± 3.45	0.86	ns
	LDHb	0.29 ± 0.13	0.36 ± 0.15	0.32	ns
	PDHx	1.1 ± 0.18	0.82 ± 0.21	0.02	*
	Pcsk1	0.07 ± 0.01	0.09 ± 0.02	0.06	ns
	SLC16A1; MCT-1	4.01 ± 1.05	3.43 ± 0.59	0.23	ns
	SLC5A1 SGLT1	88.07 ± 40.36	86.47 ± 34.17	0.94	ns
	SLC2A2; GLUT2	26.22 ± 8.74	23.57 ± 7.84	0.55	ns
	SLC2A5; GLUT5	81.18 ± 17.04	78.96 ± 27.51	0.85	ns
Jejunum	*G6Pc*	1.93 ± 1.48	2.79 ± 1.44	0.33	ns
	HK3	0.009 ± 0.002	0.008 ± 0.003	0.23	ns
	KHK	11.82 ± 3.47	14.5 ± 3.51	0.21	ns
	LDHa	14.24 ± 8.44	30.19 ± 16.78	0.05	*
	LDHb	0.041 ± 0.007	0.039 ± 0.02	0.87	ns
	PDHx	0.47 ± 0.11	0.43 ± 0.09	0.49	ns
	SLC16A1; MCT-1	2.73 ± 0.53	3.09 ± 0.42	0.23	ns
	SLC5A1 SGLT1	50.58 ± 13.28	53.04 ± 25.28	0.82	ns
	SLC2A2; GLUT2	15.5 ± 2.62	16.72 ± 5.61	0.61	ns
	SLC2A5; GLUT5	19.41 ± 5.05	24.54 ± 6.44	0.14	ns
Ileum	*G6PC*	4.19 ± 4.87	0.49 ± 0.60	0.12	ns
	HK3	0.19 ± 0.09	0.08 ± 0.09	0.06	ns
	KHK	62.36 ± 43.71	13.11 ± 15.08	0.04	*
	LDHa	227.9 ± 214.1	262.9 ± 150.9	0.75	ns
	LDHb	5.31 ± 3.25	4.79 ± 3.62	0.79	ns
	PDHx	4.95 ± 3.86	9.09 ± 6.94	0.23	ns
	SLC16A1; MCT-1	32.96 ± 28.98	34.87 ± 18.76	0.89	ns
	SLC2A2; GLUT2	65.71 ± 56.17	7.52 ± 9.12	0.05	*
	SLC2A5; GLUT5	65.13 ± 67.53	10.92 ± 12.72	0.11	ns
Colon	*Gcg*	5.54 ± 2.39	5.07 ± 3.09	0.76	ns
	LDHa	13.96 ± 14.05	21.47 ± 16.24	0.35	ns
	LDHb	1.22 ± 0.62	0.79 ± 0.49	0.17	ns
	Pcsk1	11 ± 2.79	14.39 ± 4.97	0.13	ns
	SLC16A1; MCT-1	1.85 ± 1.42	8.76 ± 4.88	0.005	**

Means and statistical differences between WD and WD-GP mRNA levels determined by unpaired, two-tailed student t-test with Welch's correction if group numbers differed. Data are shown as mean ± SD. *, $p<0.05$, **$p<0.01$.Sig.: significance. ns: not significant.

2 Supplementary Figures

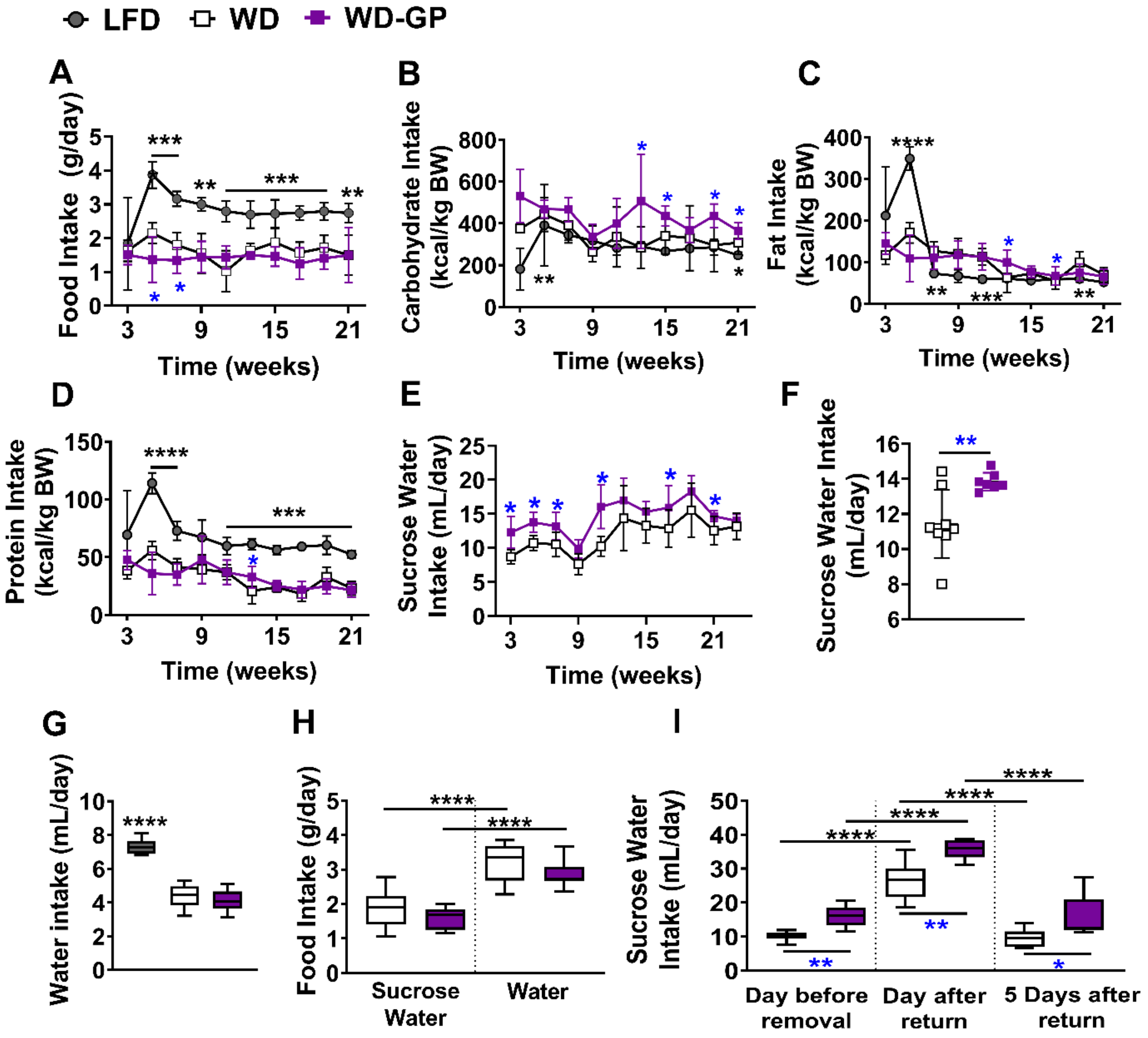
LFD
WD
WD-GP
A
Food Intake (g/day)
Time (weeks)
B
Carbohydrate Intake (kcal/kg BW)
Time (weeks)
C
Fat Intake (kcal/kg BW)
Time (weeks)
D
Protein Intake (kcal/kg BW)
Time (weeks)
E
Sucrose Water Intake (mL/day)
Time (weeks)
F
Sucrose Water Intake (mL/day)
G
Water intake (mL/day)
H
Food Intake (g/day)
Sucrose Water
Water
I
Sucrose Water Intake (mL/day)
Day before removal
Day after return
5 Days after return

Supplementary Figure 1. GPs increased caloric intake from sucrose water but not food. (A) Mean daily food consumption (g/day) at indicated week of diet intervention. Caloric intake per kg of BW from **(B)** carbohydrates, **(C)** fats, and **(D)** protein over the diet-intervention (n=6-8/group). **(E)** mean daily consumption of sucrose water (mL/day) at indicated week of diet intervention period, and **(F)** daily sucrose water consumption averaged from 23 weeks of diet intervention. **(G)** Mean water consumption after sucrose water was removed for two days. (n= 6-8/group). **(H)** Food intake during consumption of sucrose water vs. tap water (n= 6-8/group). **(I)** Sucrose water consumption (mL/day) the day before removal and replacement with plain water, one day after sucrose water was returned, and 5 days after sucrose water was returned. Data are presented as mean ± SD. Significant difference between three groups was determined by one-way ANOVA followed by Dunnett's multiple comparisons test using the WD group as control. Black colored asterisks indicate statistical significance between the LFD vs WD group and blue-colored asterisks indicate statistical significance between the WD vs WD-GP group. In panels E, F, H, and I, an unpaired, two-tailed, t-test with Welch's correction was used; *$p<0.05$, **$p<0.01$, ***$p<0.001$, ****$p<0.0001$. BW: Body Weight.

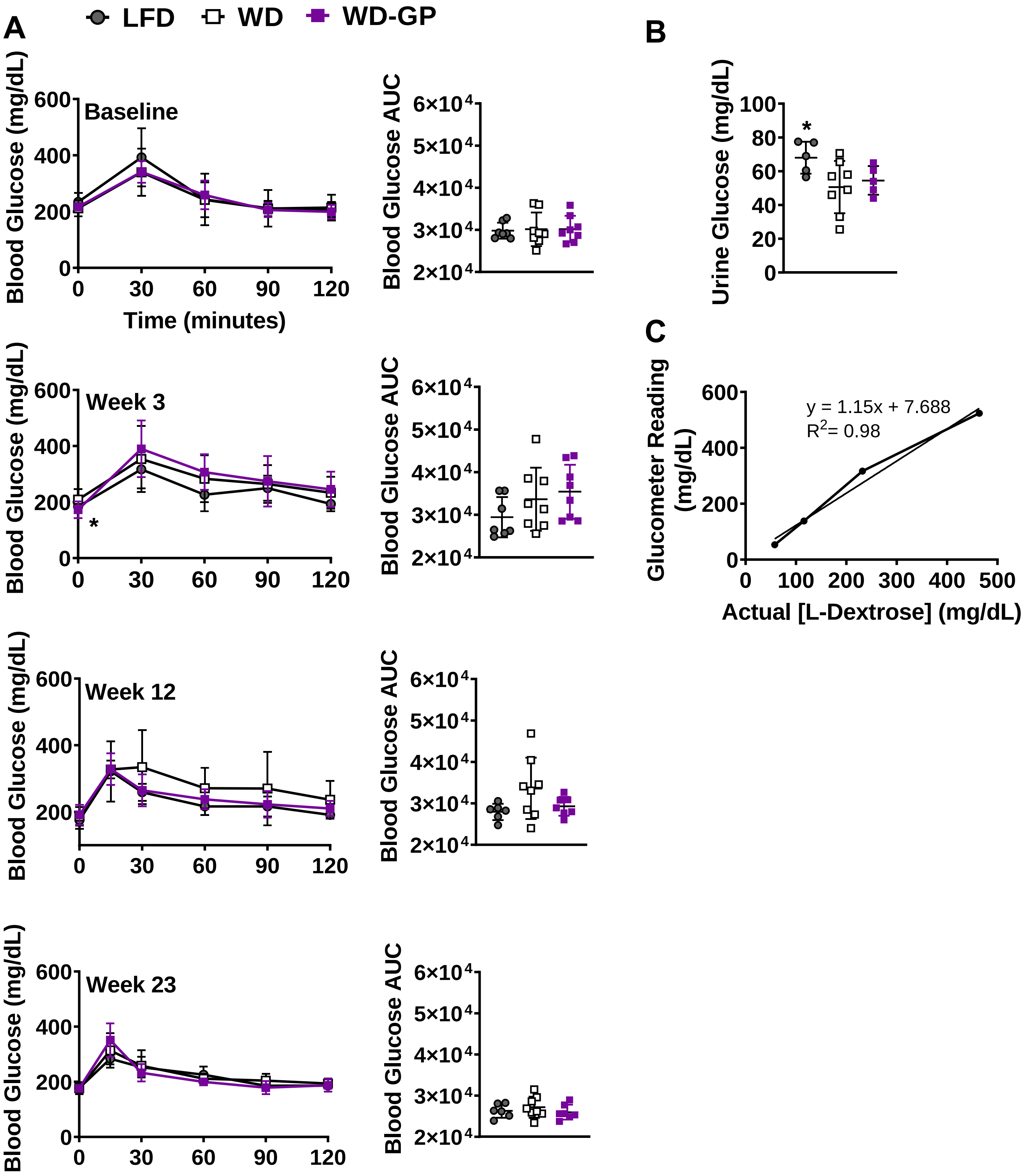
A
LFD
WD
WD-GP
Baseline
Week 3
Week 12
Week 23
Blood Glucose (mg/dL)
Time (minutes)
Blood Glucose AUC
*
B
Urine Glucose (mg/dL)
*
C
y = 1.15x + 7.688
R2= 0.98
Glucometer Reading (mg/dL)
Actual [L-Dextrose] (mg/dL)

Supplementary Figure 2. Butter-based WD did not promote hyperglycemia. (A) Oral glucose tolerance test (OGTT) results at baseline and after 3, 12 and 23 weeks of diet-intervention. Left panels: blood glucose over the 120-minutes. Right panels: mean area under the curve (AUC) data from individual mice. **(B)** Unfasted urinary glucose levels measured using a glucometer. **(C)** Correlation of glucose concentration measured by glucometer vs. dilutions of L-dextrose used for standard curve providing validation for use of glucometer to measure urinary glucose. Data are presented as mean ± SD. Significant differences between diet-group was determined by one-way ANOVA followed by Dunnett's multiple comparisons test using the WD group as control; $^{*}p<0.05$, $^{**}p<0.01$, $^{***}p<0.001$, $^{****}p<0.0001$.

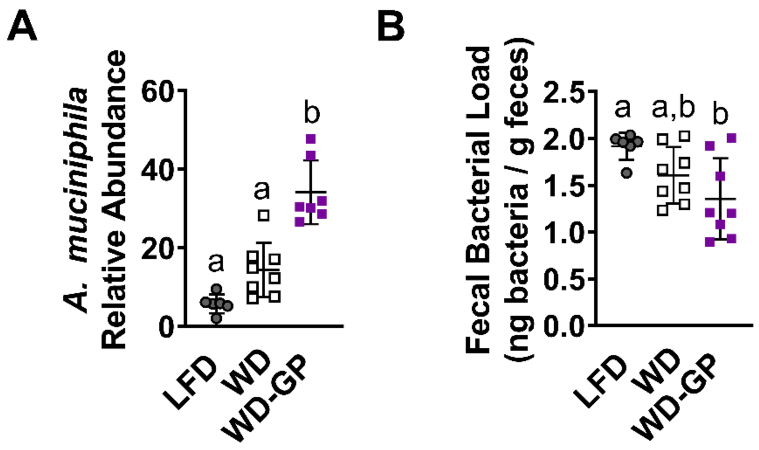

Supplementary Figure 3. GP supplementation induced a bloom of *Akkermansia muciniphila*. **(A)** Abundance of *A. muciniphila* in fecal samples relative to total bacteria and archaea, and **(B)** total fecal bacterial and archaeal load at week 8 of diet-intervention. Data are presented as mean ± SD. Different letters denote significant differences between diet-groups as determined by one-way ANOVA followed by Tukey's multiple comparisons test.
